# MYO1B in human disease: an actin-based motor linking membrane trafficking to oncogenic signaling, metastasis, and vascular aging

**DOI:** 10.3389/fonc.2026.1795888

**Published:** 2026-03-25

**Authors:** Yunxin Li, Jiaqi Zhang, Xin Gao, Feilong Zhou, Tianyi Chen, Weijie Zhao, Xinhao Li, Yewei Zhang

**Affiliations:** General Surgery Department, The Second Affiliated Hospital of Nanjing Medical University, Nanjing, China

**Keywords:** actin cytoskeleton, HNSCC, membrane trafficking, metastasis, MYO1B, oncogenic signaling

## Abstract

Myosins move along actin filaments by breaking down adenosine triphosphate (ATP) and using the energy it gives out. The superfamily presently has more than 35 classes. Mammals have the most frequent and well-preserved kind of myosin, which is class I (Myo1). People commonly group these isoforms by the morphology of their tails: long-tailed (like MYO1B, C, D), short-tailed, and tail-less. MYO1B is different from the others in that it has a lengthy tail but no second actin-binding domain. MYO1B is not evenly spread out in the body. Instead, it is mostly present near the edges of actin-rich plasma membranes and throughout the endolysosomal system, which includes early endosomes and lysosomes. It is vital for numerous bodily activities because it is located in several places, like the brain, heart, liver, and kidneys. This article brings together recent research on MYO1B’s structure, signaling pathways, and disease-causing effects. Its goal is to be a complete source for future study.

## 1 Introduction

Dynamic remodeling of the actin cytoskeleton is vital for eukaryotic cell physiology. Myosins, a superfamily of adenosine triphosphate (ATP)-dependent motor proteins, help in this remodeling by turning chemical energy into mechanical force along actin filaments (1). There are more than 35 distinct types of myosin in mammals, and they share a similar mode of operation within the motor domain. Muscle contraction is caused by sarcomeric myosins, while non-muscle isoforms control critical cellular activities like intracellular trafficking, signal transmission, polarity creation, and cytokinesis. As a result, problems with myosin are the main cause of significant diseases in people. For example, some mutations cause hereditary deafness (e.g., MYO7A and MYO15A) and hypertrophic cardiomyopathy (HCM) (e.g., MYO2), while aberrant expression patterns are associated with tumor invasion and metastasis.

Myosin I is the oldest and most frequent kind. It has an N-terminal motor domain, an IQ-motif neck, and a C-terminal tail. The tail domains of isoforms are utilized to put them into groups based on their structure (2–4). MYO1B is a unique “short-tailed” version that does not feature a second actin-binding domain or protein-interaction motifs (like SH3) (5, 6). Instead, it directly targets membrane systems with a tail portion that binds to phosphoinositides with great affinity. MYO1B is found in many vertebrate tissues (5), including the brain, heart, and liver, and it is important for cells to stay healthy (7). It moves to places in the plasma membrane that are rich in actin and the surfaces of organelles, like early endosomes and lysosomes, at the subcellular level (8–10). High-resolution imaging reveals that MYO1B is localized within dynamic membrane ruffles (9), indicating its role as a mechanosensitive anchor that connects membrane tension to cytoskeletal deformation (11–23). MYO1B controls a number of processes, such as phagocytosis, neurite outgrowth, and mechanotransduction. This study consolidates current insights into the structural basis and mechanochemical modulation of MYO1B at the membrane–actin interface, investigating its functional flexibility in physiological contexts and its potential as a therapeutic target in associated illnesses.

## 2 Structure

**Figure 1:**
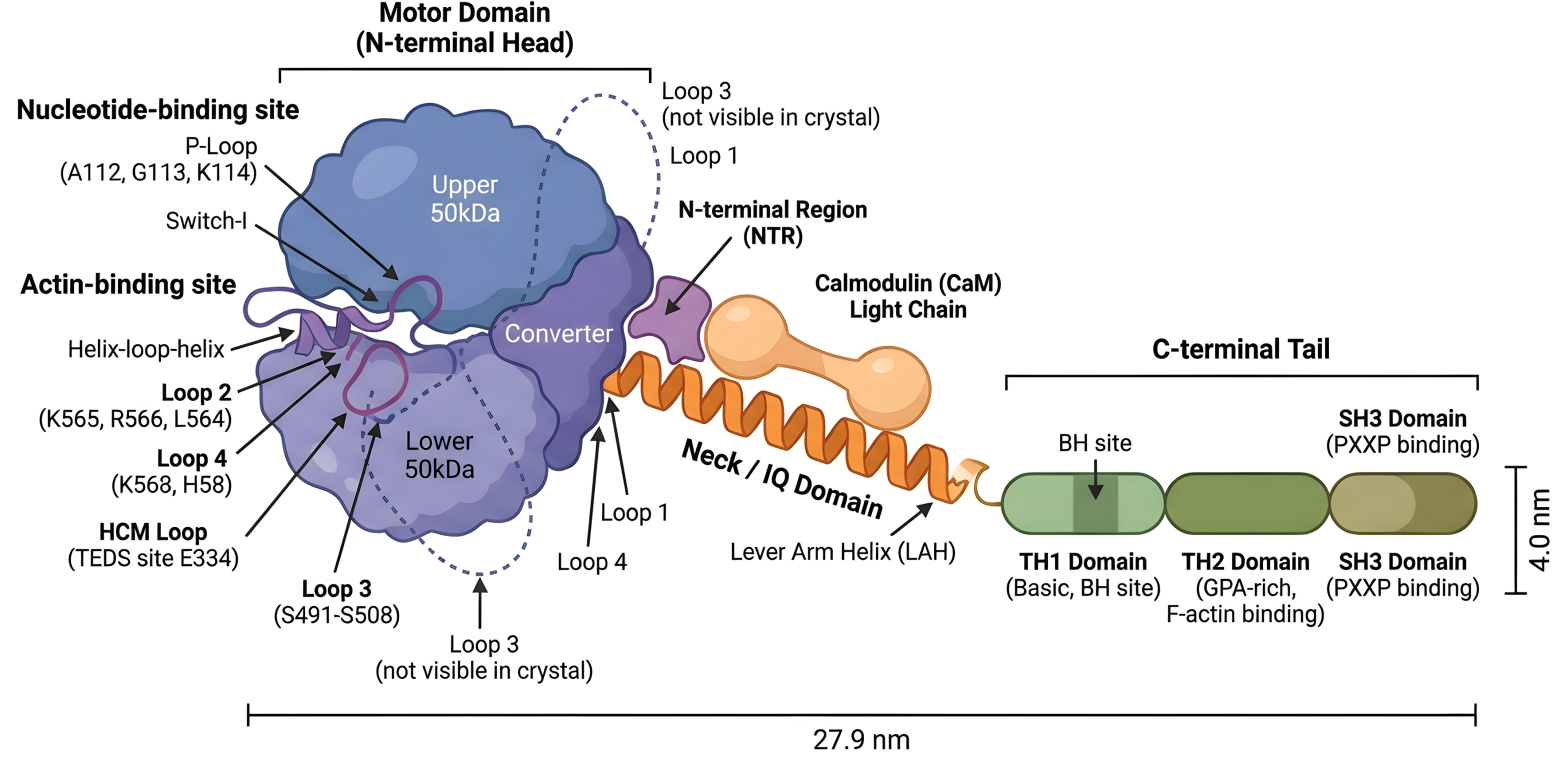
Domain architecture and key functional motifs of Myo1b. The N-terminal motor domain features the nucleotide-binding pocket (P-loop, Switch-I) and actin-binding interface (Loops 2–4, TEDS site). The neck region stabilizes the lever arm via calmodulin-binding IQ motifs. The C-terminal tail includes the basic/acidic lipid-binding TH1 domain, the TH2 domain (implicated in F-actin binding), and the PXXP-recognizing SH3 domain.

Myosin I is a set of different kinds of molecular motors that are not filamentous and are made up of only one molecule. Myosin I heavy chain has a motor domain at the N-terminal end that is very well conserved, a neck region with a unique IQ motif, and a C-terminal tail domain that can alter (16, 24, 25). The globular motor domain has the catalytic ATPase site and the actin-binding interface. The neck area next to it helps with lever arm mechanics by binding regulatory light chains, which are usually calmodulin. The non-helical C-terminal tail is the most important part of how it interacts with cellular cargo (23, 26). The tail of most class I actin motors has three parts that are the same in all of them: the basic tail homolog 1 (TH1) domain, which helps the motor attach to phospholipids; the glycine/proline/alanine-rich TH2 domain; and the Src-homologous 3 (SH3) domain (27). The SH3 domain is a protein–protein interaction (PPI) module that helps ligands with a consensus PXXP motif bind to each other through a conserved hydrophobic pocket (28, 29). Even so, the MYO1B isoform has a different structure from the other members of this family. MYO1B is not like other proteins that govern actin polymerization by bringing in other proteins since it does not feature a normal SH3 domain or a second actin-binding motif. Thus, what really makes MYO1B work is its molecular motor activity and its direct link to the membrane, not its SH3 domain scaffolding (as shown in **Figure 1**).

MYO1B is mostly made up of a splice variant that has five related calmodulins: the N-term, the upper 50 kDa, the lower 50 kDa, the converter, and the lever arm domains. Images taken with an electron microscope show that MYO1B is a single-headed molecule that looks like a tadpole and is 27.9 × 4.0 nm in size (30). The actin-binding region of myosin comprises several unique parts, including the helix–loop–helix motif in the lower 50 kDa domain, loop 2, the cardiomyopathy loop (HCM loop), loop 3, and loop 4 (31, 32). The actin-binding site is made up of the actin-binding loop and the helix–loop–helix region of the lower 50 kDa subunit. This includes loop 2, the HCM loop, loop 3, and loop 4. The P-loop and Switch I are parts of the nucleotide-binding site. Three residues in the P-loop (A112, G113, and K114) make another turn in the nearby helix, which blocks the location where nucleotides bind. No other myosin structures have this P-loop shape (33).

The motor structural domain and the lever arm helix (LAH) are both part of MYO1B crystallin. The initial calmodulin is attached to the IQ motif when there are no nucleotides present. The MYO1B structure has a very interesting feature: the N-terminal region (NTR), which is a crucial part of myocardin force sensing, is in a shape that is not seen in other myosin structures. The MYO1B NTR is situated in a hydrophobic pocket between the motor and the LAH, engaging with the first calmodulin light chain (33, 34). Therefore, it is positioned in a way that may allow it to transmit the position of LAH to the region where the nucleotide binds. The MYO1B NTR is in a hydrophobic pocket between the motor and the LAH. It interacts with the first calmodulin light chain. Therefore, it is located in a position where it may influence the nucleotide-binding site in relation to the LAH.

Loop 1 lies in the surface loop between the nucleotide binding area of MYO1B and Switch I. It changes actin affinity, ATPase activity, and nucleotide routes (35). The actin interface of loop 2 is mostly made up of basic amino acids that are the same in two myosins (K565 and R566) and the nearby L564 (36), which is not conserved. Loop 3 (S491–S508) is much longer than other members of the myosin I family, although you cannot see it in the myosin 1b crystal structure. The TEDS site at E334 in the CM loop is very stable and has a T, E, D, or S residue in practically all myosin proteins. It is also critical for controlling the ATPases of various myosin I isoforms (26, 37).

Both MYO1B and Myo1A need the BH site in the tail TH1 structural domain to be in the right place (38). The MYO1B tail has three distinct structural domains: TH1, TH2, and SH3. *In vitro*, the TH2 structural domain has been demonstrated to work as an F-actin binding (39). The SH3 structural domain is located in the C-terminal tail of MYO1B, and myosin IB has been shown to interact with proteins that control the actin cytoskeleton in other animals.

## 3 Function

MYO1B is a crucial regulator of filamentous actin (F-actin) assembly in the trans-Golgi network (TGN) and functions as an actin depolymerizing enzyme that influences actin network organization and the cytoskeleton (40). The actin cytoskeleton exhibits significant dynamism in the lamellipodium, where membrane extension is facilitated by actin polymerization. MYO1B colocalizes with F-actin in actin waves and at the apex of mature macro pinocytic cups (38). MYO1B colocalizes with F-actin in the actin wave and is situated at the apex of the mature giant erythrocyte cup. MYO1B and sorting nexin 27 (SNX27) are localized near the brush border beside MUC17 (41).

### 3.1 Promoting the formation of the trans-Golgi network

TGN serves as a principal protein sorting hub at the intersection of cellular and cytosolic pathways. Increasing evidence indicates that the actin cytoskeleton is involved in the membrane trafficking of the TGN (42), with numerous proteins related to the actin system now localized to this organelle (43–45). A MYO1B pool frequently resides near F-actin foci and the CI-mannose-6-phosphate receptor (MPR)-positive membranes within the TGN area (46). In the TGN area, the MYO1B reservoir is typically closely associated with F-actin foci and MPR-positive membranes (47). MYO1B is a mechanosensitive motor protein that interlinks load-bearing actin filaments. This protein is more proficient in sustaining and regulating cortical tension than at facilitating transport. MYO1B is a crucial modulator of F-actin assembly in the TGN area. It connects actin assembly with the TGN membrane by actively attaching and guiding polymerized F-actin bundles to it, producing forces that cause TGN membrane deformation. The force produced by MYO1B is crucial for preserving the steady-state distribution of MPRs and for linking or organizing F-actin foci on the TGN (46).

MYO1B is crucial for the tubular transport of cargo from the TGN to the Golgi apparatus and governs membrane tubule elongation through endosome sorting (10, 46). A compelling hypothesis about the mechanochemical characteristics of MYO1B is that MYO1B–F-actin foci regulate TGN membrane tension, consequently facilitating membrane deformation (48, 49). Furthermore, the MYO1B motor structural domain stabilizes freshly polymerized F-actin, resulting in the creation of F-actin foci.

**Figure 2:**
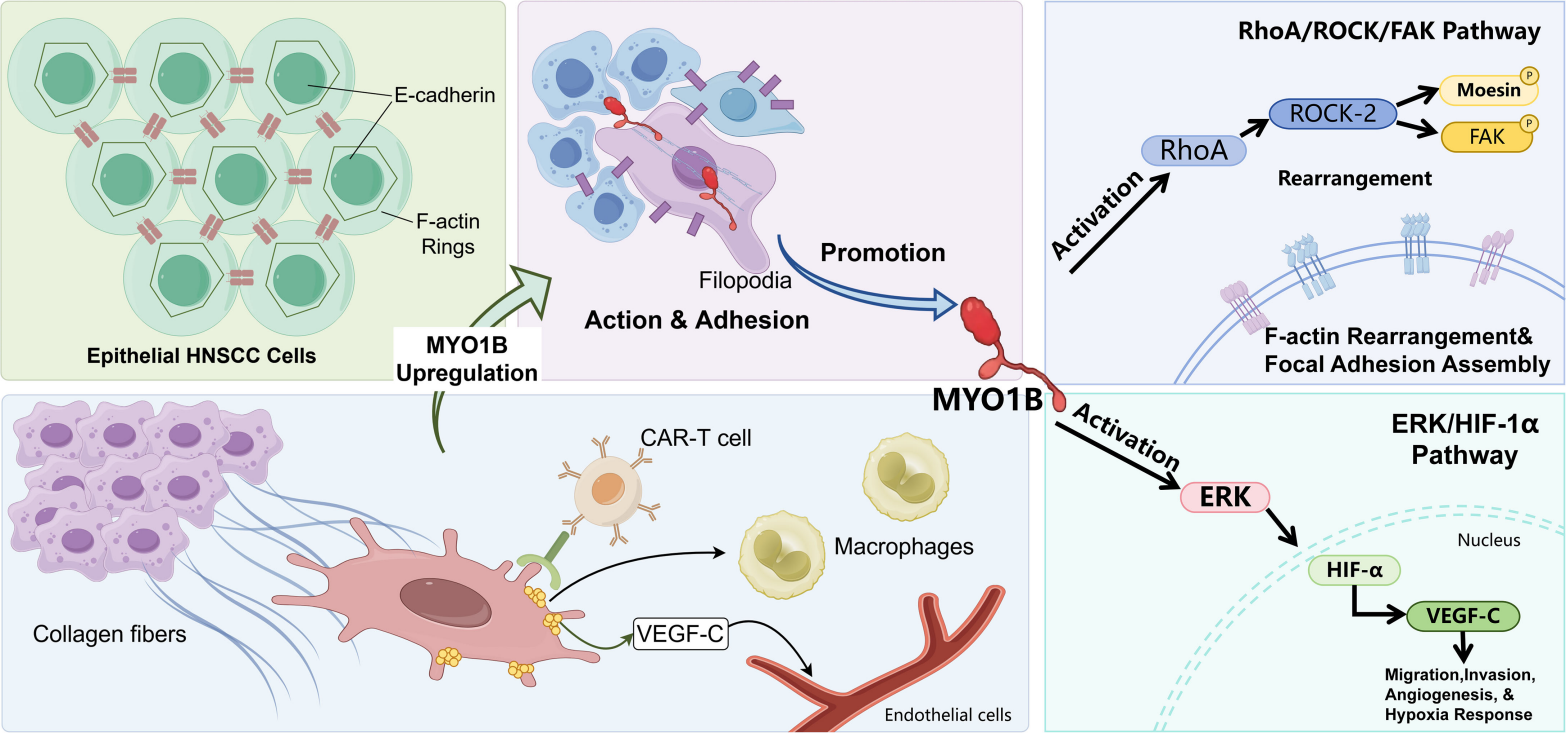
Model of Myo1b’s role in TGN tubular carrier formation. Myosin 1b promotes post-Golgi carrier formation by regulating actin assembly and membrane remodeling of the trans-Golgi network.

MYO1B on the TGN governs the trafficking of the CI-MPR to endosomes, facilitates the exocytosis of β-hexosaminidase, and mediates the transport of p75 to the plasma membrane. MYO1B depletion reduces interactions between F-actin foci and MPR membranes (50–52). The egress of MPR and p75 is contingent upon a functional actin cytoskeleton. The secretion of MPR from the TGN relies on the development of tubular carrier precursors, which extend and bifurcate to create the post-Golgi network (53, 54). The reduction of MYO1B inhibits TGN cargo egress, disrupting Golgi complex homeostasis and altering its shape (as shown in **Figure 2**).

Members of the Rho superfamily of small GTPases are essential regulatory molecules for cell migration, mediating the activation of signaling pathways linked to cytoskeletal remodeling, with Ras homolog family member A (RhoA), CDC42, and RAC1 as prominent members (55). MYO1B facilitated the migration, invasion, and motility of colorectal cancer (CRC) cells via targeting RhoA. The Rho-associated coiled-coil containing protein kinase 2 (ROCK2)/LIM domain kinase (LIMK)/Cofilin axis is a recognized signaling route linked to F-actin rearrangement, and its activation facilitates F-actin reorganization. The overexpression of MYO1B increases the phosphorylation of ROCK2, LIMK, and Cofilin in SW480 cells, suggesting that MYO1B facilitates the activation of the ROCK2/LIMK/Cofilin pathway. MYO1B facilitates CRC spread by boosting F-actin reorganization and focal adhesion formation through the RhoA/ROCK/focal adhesion kinase (FAK) signaling pathway (56).

The membrane-associated mucin MUC17 in intestinal epithelial cells keeps a highly tuned system for where the apical membrane is located (57). MUC17 is a major part of the intestinal glycocalyx barrier (58). Research demonstrates that the apical localization of MUC17 is an active process, dependent on a relay recycling pathway facilitated by SNX27 sorting, MYO5B transport, and MYO1B insertion stabilization. MYO1b is very abundant at the apical brush border of intestinal epithelial cells. It helps MUC17-containing vesicles get through the dense terminal plate actin layer and into microvilli. MUC17’s cytoplasmic tail has a PDZ-binding motif, which lets sorting connector SNX27 directly recognize and bind MUC17 through its PDZ domain. MYO5B moves vesicles along actin filaments from deep cytoplasmic recirculating endosomes to the top of the cell, just below the plasma membrane. MYO1b takes control once MYO5B delivers it to the top. MYO1b breaks through the resistance of the apical dense actin network to let vesicles fuse with the apical membrane or move MUC17 into microvilli (59).

Both loss-of-function mutations in MYO5B and a deficit in UNC45A cause microvillus inclusion disease (MVID) (60) (61). Cells in the intestines that do not have MYO5B and UNC45A usually do not have microvilli, but they do have microvillus inclusions, large intracellular vesicular structures, and a lot of intracellular vesicles (62). MYO1b physically interacts with UNC45A through its specific domain. UNC45A is a molecular chaperone that helps myosin fold and come together. MYO1b draws UNC45A into intestinal epithelial cells and gathers it in the cell’s apical cortex. Without MYO1b, it is not possible to correctly place UNC45A in the apical area. When MYO1b is not present or its function is impaired, UNC45A gets mislocalized, which can lead to uncontrolled assembly or activity of apical NMIIA (63). This could cause problems with contractile force or distribution. In the end, this means that epithelial cells cannot do their jobs of properly contracting at the top and folding the tissue. The result makes it harder for the intestinal villi to grow, which is shown by the intestinal tube getting shorter and flatter.

### 3.2 Mechanochemical regulation of actin dynamics by Myo1

#### 3.2.1 Mechanochemical properties and actin dynamics

MYO1B operates as a specialized mechanosensitive motor that regulates actin network organization via tension-dependent processes (48). In contrast to traditional motors that mainly facilitate movement, MYO1B has a “catch-bond” behavior, wherein the duration of its binding to F-actin markedly extends under applied force (64). This force-dependent anchoring enables MYO1B to apply strain on actin filaments, thus affecting their polymerization kinetics (65). Single-molecule characterization demonstrates that the translocation of F-actin along MYO1B assemblies not only diminishes the polymerization rate but also actively facilitates filament debranching and inhibits the emergence of new dendritic branches. Thus, MYO1B functions as a mechanical actin depolymerase, utilizing force to regulate cytoskeletal structure. The interaction cycle comprises two separate mechanical phases: an initial phase related to inorganic phosphate (Pi) release, succeeded by a second stroke connected with adenosine diphosphate (ADP) release, which determines the duty ratio and force sensitivity (66, 67).

#### 3.2.2 Structural determinants of the actin interface

The arrangement of surface loops in the motor domain determines the strength and specificity of the MYO1B–actin interaction. The HCM loop (residues T328–S337) is an important aspect of the process of high-affinity binding (68). There are hydrophobic clusters that hold it in place, and one of these is Actin-Y337 (69). The E334 residue in MYO1B interacts with the K336 and Y337 residues in Actin (70). This is different from myosin II, where homologous residues do not touch the actin surface. The regulatory implications of this unique HCM loop–nucleotide interaction remain a critical area for more structural elucidation.

#### 3.2.3 Adjacent surface loops further refine this interface

Loop 2 is a member of the myosin I family. It is stiff because it has conserved proline residues (P568–P567) that are peculiar to that category. This means that it does not alter form much when it attaches. Loop 3, on the other hand, goes through an order–disorder transition and becomes structured when it interacts with actin to make hydrophobic contacts around Y91. Loop 4 adds a weak, modulatory hydrophobic network that is not powerful enough to decide where in the cell it should go on its own (68), but it probably collaborates with the tail area to make the filament more particular. The HCM loop, loop 2, and loop 4 all work together to keep the actin-binding interface from turning on too quickly when the system is in the auto-inhibited state (71, 72).

#### 3.2.4 Force sensitivity and calcium regulation

The light-chain binding domain (LCBD) of MYO1B is what makes it mechanosensitive (73–76). It acts as a lever arm. The force-dependent dissociation rate, and consequently the attachment duration, is proportional to the length of the LCBD (32, 49), corroborating a concept in which LCBD rotation represents the force-sensitive transition of the working stroke. The quantities of calcium inside the cell strictly control this mechanism (49). Calmodulin (CaM) stabilizes the LCBD by acting as both a structural subunit and a switch that controls it. When calcium binds to CaM, it makes it less likely to connect to the MYO1B heavy chain. This causes one or more CaM molecules to come apart from the neck region. This separation of ATPase activity from motility essentially ends actin attachment (77). On the other hand, adding exogenous calmodulin can stop this inhibition, creating a reversible, Ca^2+^-sensitive way to control MYO1B motility (6, 78, 79).

#### 3.2.5 Cellular implications of MYO1B activity

The physiological effects of MYO1B go beyond just moving things around. Studies of overexpression reveal that MYO1B changes the organization of the F-actin network not by changing the overall G-actin to F-actin ratio, but by changing the structure of the filaments that are already there. This reconfiguration happens because the motor domain and the lipid-binding tail are both active at the same time (80). This efficiently links actin to membranes or other parts of the cytoskeleton (81). Some class I myosins use SH3 domains to bring in polymerization machinery, whereas MYO1B-dependent remodeling seems to depend on its own actin-binding motifs and mechanochemical characteristics (82). For example, the higher viscosity of the cytoplasm seen in certain cells when MYO1B is overexpressed is due to the heavy chain’s actin-binding sites making more cross-links, not new polymerization. Thus, MYO1B is a dual-function regulator: it is a motor that creates tension and speeds up depolymerization, and it is a dynamic cross-linker that keeps the cytoskeleton strong under stress.

### 3.3 MYO1B-driven membrane remodeling and tubulation

MYO1B is a key mechanical connection between lipid membranes and the actin cytoskeleton (83). It makes organelles change shape and move (84). MYO1B leverages its intrinsic catch-bond property to provide enough force to push tubular membrane extensions out of PI(4,5)P_2_-rich bilayers along F-actin bundles at normal densities (83). MYO1B-mediated tubulation acts by itself, without tip clustering, and only needs a few motors to start pressing on the membrane. Non-claret disjunctional (NCD) is a kinesin-family motor, but this is not like that (85–87). At the TGN, MYO1B comes together in groups that modify the membrane’s tension (8, 49). MYO1B alters the morphology of the TGN bilayer to facilitate the growth of tubular carrier precursors through the integration of force generation and localized actin polymerization. This process indicates a sequential transport model wherein MYO1B and F-actin commence short-range membrane extraction, thereby connecting the spatial distance between the organelle surface and the microtubule network (46). Long-range microtubule-associated motors, such as kinesins, facilitate the transport of new tubules over extended distances. This demonstrates the essential role of MYO1B in initiating the process of carrier formation.

### 3.4 MYO1B and the establishment of neuronal polarity

MYO1B is a membrane-associated single-headed motor protein that binds to membranes via its carboxy-terminal domain (also termed the tail). The C-terminal tail region of MYO1B controls its membrane recruitment. This region contains a highly basic TH1 domain with a possible PH motif. This structural module enables calcium-independent docking onto anionic phospholipid bilayers, with a particular focus on phosphoinositides. The MYO1B tail can bind to both PI(4,5)P_2_ and PI(3,4,5)_3_ *in vitro*, although it has a strong preference for PI(4,5)P_2_ (88, 89). Because PI(4,5)P_2_ is much more common in the body than PI(3,4,5)_3_, MYO1B is only found in areas of the cell that are rich in PI(4,5)P_2_ (90), like filopodia, instead of the broad areas of the cytoplasm where PI(3,4,5)_3_ sensors are found (88). When it binds, MYO1B may still move about laterally in the fluid bilayer, which lets it dynamically follow phosphoinositide gradients that form during cell polarization (91). This lipid connection is limited to the C-terminal tail; in contrast to the similar isoform Myo1c, the IQ motifs of MYO1B do not engage in membrane association, highlighting a unique modular architecture for membrane interaction (92).

### 3.5 MYO1B regulation of growth cone F-actin organization and microtubule transport

An essential step in neuronal development is establishing the polarity required for axonogenesis. Actin waves, propagating along the neurite axis upon contact and deformation of the plasma membrane, are key contributors to axon formation (93, 94). The fusion of actin waves with the neurite tip correlates with neurite growth, and actin waves share similar molecular composition and dynamics with the growth cone (84, 95–97). The actin bundles in the growth cone form filamentous pseudopodia and a dense actin network that connects to the root filaments of actin bundles in the intermembrane zone of the actin wave (98).

MYO1B regulates neuronal polarization and axonal extension by limiting the expansion of the branching actin network and supporting actin bundles within the growth cone (84). Studies indicate that MYO1B controls the size of actin domains within the growth cone, the propagation of actin waves, and the stability of filopodia. MYO1B appears at higher concentrations in the growth cones of stage 2 (DIV2) and stage 3 (DIV3) neurons, localized in the cell body and along the axonal growth cone. Furthermore, the enrichment of MYO1B in the growth cone is independent of axon length in stage 4 (DIV4) neurons (99).

Research indicates that MYO1B is essential for the anterograde propagation of actin waves to the apical end of neurites, rather than their initial formation, and that both the motility activity of MYO1B and the integrity of its PH motif are necessary for this function (84). The MYO1B PH motif facilitates its association with the plasma membrane. Studies compared the percentages of neurons lacking axons, possessing a single axon, or exhibiting multiple axons in cells expressing enhanced green fluorescent protein (EGFP), EGFP-MYO1B, or EGFP-MYO1B-k966a. EGFP-MYO1B-k966a is a mutant of the PH motif that inhibits its binding to phosphatidylinositol (88). The results indicate that MYO1B’s association with the plasma membrane via its interaction with phosphatidylinositol is essential for symmetry breaking and axon formation (84).

Kinesin family member 5C (Kif5C) is a microtubule-associated motor responsible for transporting proteins associated with axonal differentiation (100, 101). The minimal motility domain of Kif5C560-EGFP is considered an early marker of axons, where actin waves associated with microtubule polymerization control the random fluctuations of Kif5C (102). MYO1B influences the random fluctuations of Kif5C from one neurite to another by regulating the anterograde migration of actin waves. MYO1B regulates F-actin organization in the growth cone by controlling the size of the p-domain containing F-actin, including the filamentous pseudopodia and the veil between filamentous pseudopodia, as well as the stability of filamentous pseudopodia (84). Because of its role in F-actin organization, MYO1B indirectly modulates microtubule distribution and microtubule-dependent transport (101).

### 3.6 MYO1B regulation of autophagy pathways and cell fate

Extensive evidence indicates that autophagy plays a crucial role in regulating cellular senescence, with several types of myosin involved in autophagy regulation (103–108). Upregulation of MYO1B expression in endothelial cells inhibits LC-II and p62 autophagosome–lysosome fusion and degradation (109). MYO1B mediates endothelial autophagy by interfering with autophagic flux, thereby regulating endothelial senescence and senescence-associated endothelial dysfunction.

In a cellular model of myocardial ischemia/reperfusion (I/R) injury, MYO1B expression decreased following I/R stimulation. Overexpression of MYO1B prevented hypoxia/reoxygenation-induced cardiomyocyte apoptosis and proliferation inhibition in H9c2 cells (110). MYO1B mediates the activation of mTORC1-S6K1 by Arg-II through promoting peripheral lysosomal localization, leading to the spatial separation and dissociation of TSC from lysosomes. This results in the excessive activation of mTORC1-S6K1 signaling associated with cellular senescence/apoptosis (111). MYO1B participates in regulating H/R-induced cardiomyocyte apoptosis and autophagy, potentially serving as an endogenous target for preventing I/R injury (101, 110).

PPIs play a crucial regulatory role in numerous vital cellular functions and biological processes. Leucine-rich repeat serine/threonine-protein kinase 2 (LRRK2) is implicated in multiple cellular processes, including autophagy (112). LRRK2 regulates autophagy through the calcium-dependent protein kinase-β (CaMKK-β)/AMP-activated protein kinase (AMPK) pathway (113). Intercellular calcium plays a key regulatory role in multiple cellular processes, including cellular senescence and autophagy. Interaction between MYO1B and LRRK2 via the tail domain of MYO1B promotes intracellular calcium elevation, thereby inhibiting autophagy flux.

AKT, also known as protein kinase B (PKB), is a serine/threonine kinase that plays a crucial role in various cellular processes, including cell growth, proliferation, and metabolism (114, 115). Hyperactive AKT is found in multiple tumors (116, 117). mTORC1, AKT, and the angiotensin homolog [phosphatase and tensin homolog (PTEN)] play important roles in regulating cellular aging and proliferation. Nuclear PTEN promotes apoptosis (118). MYO1B interacts with PTEN to regulate nuclear AKT activation and apoptosis by blocking PTEN nuclear localization in B16-F10 melanoma cells (119).

### 3.7 MYO1B and the insulin secretion pathway

Insulin transport is mediated by the actin cytoskeleton, and several members of the myosin superfamily of actin-associated molecular motors have been shown to participate in insulin transport (120–125). Insulin-induced AKT activation depends on phosphoinositide 3-kinase and is opposed by tumor suppressor phosphatases and PTEN (119). Studies indicate that depletion of MYO1B reduces the early transport of newly synthesized insulin granules from the TGN, decreases the number of granules budding from the TGN, and delays their transport from the TGN to the plasma membrane (126).

## 4 Signaling pathway

MYO1B plays a crucial role in multiple signaling pathways. MYO1B, acting as a mediator of Arg-II-induced mTORC1-S6K1 activation—specifically, mediating Arg-II’s lysosomal redistribution and TSC-lysosomal dissociation via its MYO1B-PH domain—represents a novel mechanism regulating the mTORC1-S6K1 pathway and associated with vascular aging (127–129). In high-risk neuroblastoma (NB), macrophage migration inhibitory factor (MIF) functions as a multifunctional cytokine acting as a hormone, chaperone, and enzyme (via N-terminal proline with isomerase activity) (130–132). MYO1B is regulated by growth regulating estrogen receptor binding 1 (GREB1) in MYCN-negative neuroblastoma (MNA NB) in a MYCN-independent manner, indicating that the GREB1–MYO1B–MIF axis promotes aggressive behavior in MNA+ NB as an independent mechanism. In CRC, MYO1B overexpression promotes phosphorylation of ROCK2, LIMK, and Cofilin in SW480 cells, indicating that MYO1B activates the ROCK2/LIMK/Cofilin axis. In esophageal squamous cell carcinoma (ESCC), MYO1B activates the SNAI2/cyclin D1 pathway, driving ESCC tumorigenesis and cisplatin cytotoxicity, indicating MYO1B as a potential therapeutic target in patients with ESCC (133). In the intracellular milieu of acute myeloid leukemia (AML) cells, the highly produced AC024896.1 functions as a competitive endogenous RNA (ceRNA), specifically sequestering and binding to the microRNA (miRNA) miR-363-3p. The link stops miR-363-3p from stopping MYO1B, which causes MYO1B protein to build up too much. This protein is an important effector molecule (134). This buildup directly increases the speed at which leukemia cells grow, move, and invade other cells, while also stopping scheduled cell death, which makes the disease worse (as shown in **Table 1**).

**Table 1:** Signaling pathways regulated by MYO1B and their corresponding molecular mechanisms and biological effects.

Signaling mechanism	Disease/Context	Role of MYO1B	References
mTORC1-S6K1 pathway (Arg-II-induced)	Vascular aging	Acts as a mediator of Arg-II-induced activation; specifically mediates Arg-II’s lysosomal redistribution and TSC-lysosomal dissociation via the MYO1B-PH domain.	(127–129)
GREB1–MYO1B–MIF axis	High-risk neuroblastoma (NB)	Regulated by GREB1 in a MYCN-independent manner; cooperates within the GREB1–MYO1B–MIF secretory axis to promote aggressive behavior in MNA+ NB.	(130–132)
ROCK2/LIMK/Cofilin axis	Colorectal cancer	Overexpression promotes the phosphorylation of ROCK2, LIMK, and Cofilin, thereby activating the axis in SW480 cells.	(56)
SNAI2/Cyclin D1 pathway	Esophageal squamous cell carcinoma (ESCC)	Activates the SNAI2/cyclin D1 pathway, driving tumorigenesis and influencing cisplatin cytotoxicity.	(133)
AC024896.1/miR-363-3p/MYO1B axis (ceRNA mechanism)	Acute myeloid leukemia (AML)	Functions as a downstream effector; its protein accumulation (due to AC024896.1 sequestering miR-363-3p) accelerates cell proliferation, migration, and invasion while inhibiting apoptosis.	(134)

## 5 Disease

### 5.1 Cancer

#### 5.1.1 Head and neck squamous cell carcinoma

Head and neck squamous cell carcinoma (HNSCC) ranks as the sixth most common malignant tumor globally (135). MYO1B, a member of the myosin family, was identified as being upregulated in HNSCC through a network-based bioinformatics meta-analysis.

Immunohistochemistry (IHC) revealed that abnormal MYO1B expression correlated with lymph node metastasis (LNM) in patients with HNSCC (*n* = 31, *p* = 0.0320). Filopodia, platypodia, and invaginations/podosomes are known to participate in cancer cell migration, and MYO1B has been reported to localize to the membranes of these protrusions (88, 136). Knockdown of the MYO1B gene suppressed migration and invasion of HNSCC cells by reducing the formation of large protrusions on the cell membrane, independent of major epithelial–mesenchymal transition (EMT) transcription factors (137, 138). Downregulation of MYO1B in human HNSCC cells reduced LNM (138). MYO1B expression in primary HNSCC tissue represents a potential diagnostic biomarker for predicting LNM and subsequent prognosis in patients with HNSCC.

To date, most studies indicate that miRNAs can bind to specific sequences within the 3′UTR, 5′UTR, and coding regions, as well as within the promoter regions of target mRNAs, to induce translational repression, mRNA degradation, and mRNA cleavage (139). Analysis of the miRNA expression signature of HNSCC based on RNA sequencing showed that dual strands of pre−miR−145 (miR−145−5p, guide strand; and miR−145−3p, passenger strand) were significantly reduced in cancer tissues (140–145). MYO1B is also regulated by miR-363, while miR-16 is induced by the HPV 6-E2015 oncoprotein and contributes to the migratory capacity of head and neck cancer cells (146). The passenger strand of miR−145 acted as an antitumor miRNA through targeting MYO1B in HNSCC cells (147).

Studies have demonstrated that MYO1B enhances the phosphorylation levels of the ATM (ataxia-telangiectasia mutated) protein (148). The ATM enzyme is in charge of much of the DNA damage response (DDR). When radiation therapy damages DNA, MYO1B, which is highly expressed, quickly activates ATM through the PI3K/AKT pathway. This cycle is the initial stage in the process of repairing DNA damage that occurs afterwards. Results from experiments show that cells that overexpress MYO1B have far less nuclear damage from radiation. However, when you pull down MYO1B, γ-H2AX levels increase a lot. This indicates that DNA repair is not occurring and damage remains unrectified. MYO1B makes important pluripotency transcription factors like SOX2 and OCT4 work better. Increasing the levels of these chemicals keeps HNSCC cells in a condition similar to stem cells. Tumor stem cells are usually in a resting stage (G0) and can easily generate more of themselves. This procedure makes them naturally resistant to radiation, which usually hurts cells that are dividing quickly. In short, high levels of MYO1B expression turn on the PI3K/AKT pathway, elevate SOX2/OCT4 levels to make cells more pluripotent, promote ATM phosphorylation, speed up DNA repair, and help tumor cells avoid damage from radiation. This research indicates that MYO1B may serve as a viable therapeutic target for improving radiation sensitivity in HNSCC.

**Figure 3:**
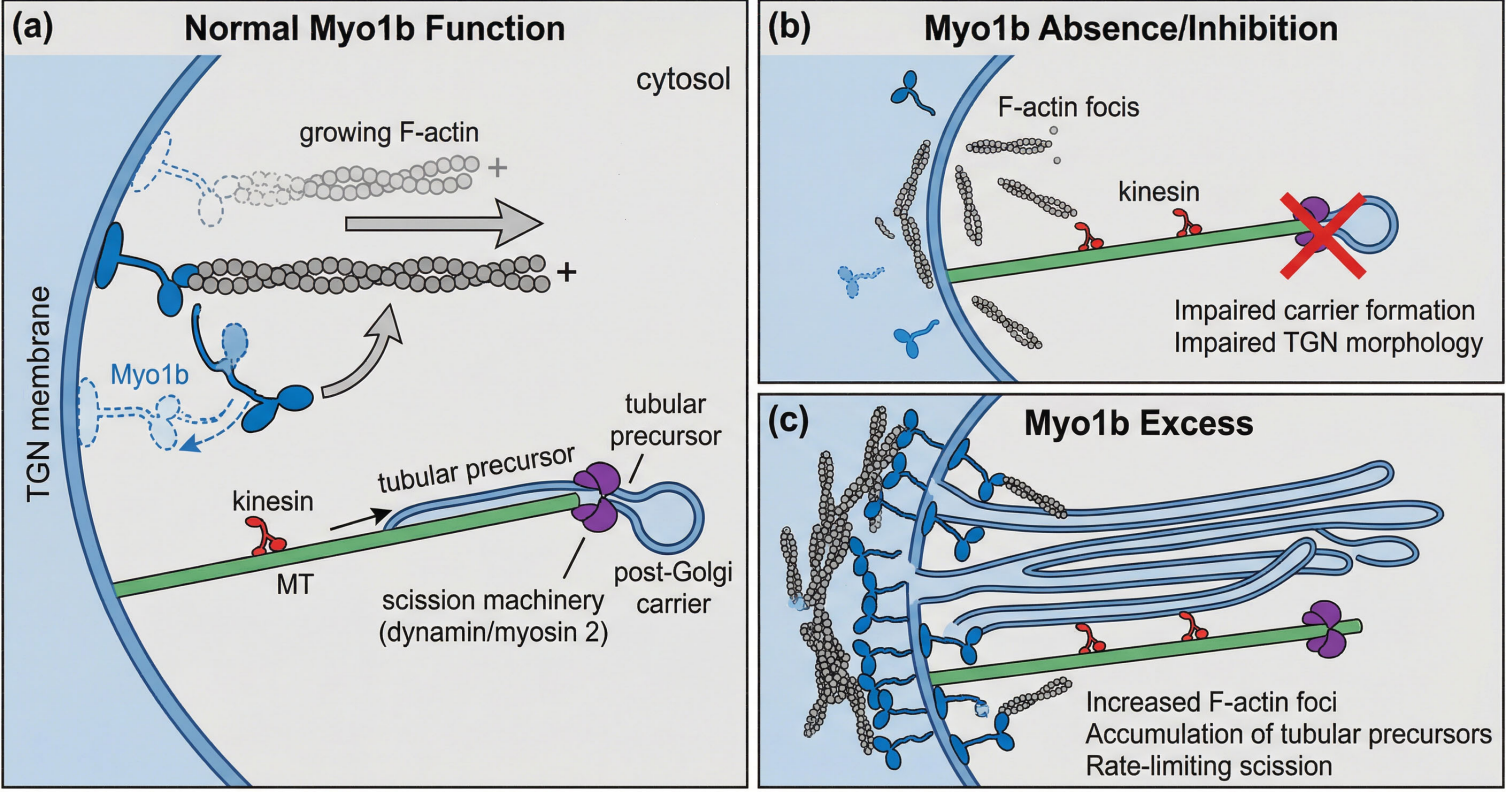
Schematic of MYO1B signaling and TME modulation in HNSCC metastasis. MYO1B upregulation promotes EMT and filopodia formation by disrupting E-cadherin junctions and remodeling F-actin. Mechanistically, MYO1B drives cytoskeletal dynamics via the RhoA/ROCK-2/FAK axis (enhancing focal adhesion assembly) and promotes angiogenesis and hypoxia responses via the ERK/HIF-1α/VEGF-C axis. These pathways facilitate invasion through the extracellular matrix and modulate the tumor microenvironment, including interactions with endothelial and immune cells. Model for Myo1b function at the TGN. **(a)** Myo1b drives F-actin-dependent membrane tubulation and post-Golgi carrier formation. **(b)** Myo1b loss or inhibition disrupts TGN actin organization and impairs carrier biogenesis. **(c)** Myo1b excess promotes tubular precursor accumulation, consistent with scission becoming rate-limiting.

Targeting MYO1B impairs tumorigenesis via inhibiting the SNAI2/cyclin D1 signaling in ESCC (133). Oral cancer is insidious in its early stages and is characterized by high invasiveness, early LNM, rapid growth, and a high metastasis rate (149) (150). MYO1B may be mainly related to metastasis, angiogenesis, hypoxia, and differentiation. A positive correlation between MYO1B and the infiltration of macrophages, B cells, and dendritic cells was presented. MYO1B might have a close relationship with SMAD3, which may be enriched in the Wnt signaling pathway. MYO1B suppression markedly inhibited the proliferation, invasion, and metastasis abilities of both Arecoline-transformed oral cells and oral cancer cells (151) (as shown in **Figure 3**).

Oral tongue squamous cell carcinoma (OTSCC) represents a distinct subgroup of head and neck cancers characterized by high aggressiveness, recurrence, and metastasis rates (152–155). Transcriptome sequencing (RNA-seq) analysis revealed that MYO1B overexpression correlates with treatment failure in early-stage (T1 and T2) OTSCC, offering a clinical tool to distinguish early-stage patients from those with occult nodular metastases (156).

#### 5.1.2 Esophageal squamous cell carcinoma

ESCC is the most prevalent type of esophageal cancer and ranks as the sixth leading cause of esophageal cancer-related deaths globally (157). Numerous miRNAs exhibit differential expression in ESCC, contributing to its pathogenesis through their activity as oncogenes or tumor suppressors (158). Thirty potential oncogenic targets regulated by miR-145-3p were identified in ESCC cells. Among these targets, dehydrogenase/reductase family member 2 (DHRS2) and MYO1B were selected for in-depth investigation to elucidate their functional roles in ESCC cells. Dual luciferase reporter assays confirmed direct regulation of DHRS2 and MYO1B by miR-145-3p in ESCC cells. Abnormal expression of DHRS2 and MYO1B was detected in clinical ESCC specimens, and their overexpression enhanced the invasiveness of cancer cells (159) These findings indicate that aberrantly expressed MYO1B is associated with cancer cell aggressiveness and metastasis. MYO1B may be a novel diagnostic and therapeutic target for patients with ESCC.

SNAI2 is a member of the conserved transcription repressor SNAIL family, participating in numerous critical biological regulatory processes such as EMT, cellular reprogramming, differentiation, morphogenesis, and tumorigenesis (160–163). The distribution of MYO1B protein is regulated by serine/arginine-rich splicing factor 1 (SRSF1), shifting from the cell membrane to cytoplasmic dispersion, thereby influencing cancer cell behavior (164). Cyclin D1, an oncogene (165, 166), also serves as an effector of SNAI2 in ESCC cells (167).

Bioinformatics analysis and mechanistic studies indicate that SNAI2 is a key downstream effector of MYO1B. Inhibition of MYO1B downregulates SNAI2 expression, thereby suppressing the SNAI2/cyclin D1 pathway. Furthermore, selective inhibitors of cyclin D1 activation can reverse the invasive phenotype of ESCC cells induced by siMYO1B cells overexpressing snai2. MYO1B positively correlates with SNAI2 and cyclin D1 in ESCC samples, and high SNAI2 expression is associated with poor prognosis in patients with ESCC. These findings demonstrate that MYO1B activates the SNAI2/cyclin D1 pathway, driving ESCC tumorigenesis and cisplatin cytotoxicity, suggesting MYO1B as a potential therapeutic target for patients with ESCC (133).

Several E3 ligases, such as mouse double minute 2 (MDM2), β-TrCP1, FRXL14, FBXW1, CHIP, and ankyrin repeat and SOCS box containing 13 (ASB13), have been reported to target SNAI2 for ubiquitination and degradation (168, 169). Notably, only ASB13 is a true E3 ligase that targets endogenous SNAI2 protein for ubiquitination. Whether ASB13 participates in regulating the MYO1B/SNAI2 pathway and modulating ESCC invasiveness warrants further investigation.

#### 5.1.3 Prostate cancer

Prostate cancer is the most common cancer type among men worldwide. Treatment management depends on whether the cancer is hormone-resistant or hormone-sensitive. Hormone-resistant prostate cancer exhibits distinct surface proteins that confer different migratory capabilities and interactions with organs and tissues. In contrast, hormone-sensitive prostate cancer migrates and frequently metastasizes.

Examination of myosin expression in prostate cancer cell lines revealed elevated levels of MYO1B in cells with high metastatic potential and in metastatic tumors (170). Myeloid-associated differentiation markers, myosin 1b, and phosphatidylinositol-4-phosphate-5-kinase type 1 alpha are implicated in metastasis (171). Their knockdown altered the expression of key regulators of endothelial–mesenchymal transition, invasion, motility, and migration. In primary prostate tumors, these genes could be an indicator for future metastasis into lymph nodes.

#### 5.1.4 Glioma

Gliomas are the most common primary brain tumors (172, 173). Alternative splicing (AS) of precursor mRNAs is a crucial post-transcriptional process determining the complexity of mammalian proteomics (174–176). SRSF1 plays a vital role in AS. Studies indicate that SRSF1 is an effective promoter of glioma cell proliferation, survival, and invasion (164).

SRSF1 is primarily driven by the transcription factor MYC and exerts oncogenic effects by controlling AS of cancer-associated genes (177–179). SRSF1 directly interacts with MYO1B pre-mRNA after transcription to determine how it is processed. SRSF1 attaches to conserved motifs on both sides of exon 23 of the MYO1B transcript. This makes it easier to add the exon in a different approach. This particular splice-switching event is a major reason why there is so much MYO1B protein. The SRSF1-regulated isoform that results from this mechanism maintains its complete structural integrity, preserving the functional competence of both its C-terminal tail and motor domains. It is very important that this structure stays intact because it gives MYO1B the framework it needs to work as a molecular scaffold, which helps PDK1 and PAK1 and other downstream signaling effectors attach and dock. Thus, this specific MYO1B isoform is transported to the plasma membrane, where it settles on moving actin-rich protrusions like lamellipodia and filopodia toward the front of tumor cells. MYO1B appears to be at risk of being misplaced or breaking down too soon in the cytoplasm without this SRSF1-mediated regulation. SRSF1-guided MYO1B pre-mRNA splicing determines cell fate through PDK1/AKT and PAK/LIMK pathways. MYO1B, which is found on the cell membrane, works as a scaffold by directly attaching to its domain and bringing PDK1 (phosphoinositide 3-kinase 1) from the cytoplasm to the cell membrane. After the membrane is enriched, PDK1 may get to its substrate, AKT. After that, PDK1 adds a phosphate group to AKT at the Thr308 location. Phosphorylation of AKT activates the kinase, starting pro-survival signaling pathways that stop apoptosis and encourage cancerous growth in glioblastoma cells. MYO1B also acts as a scaffold, bringing PAK1 (p21-activated kinase 1) to the pseudopodial structures of the cell membrane. When PAK1 is brought to the membrane, it becomes active and then phosphorylates and activates its downstream substrate, LIMK. LIMK, which is a protein that controls filaments, then phosphorylates cofilin. The main job of cofilin is to cut actin filaments (F-actin) to speed up turnover. When LIMK phosphorylates cofilin, it stops working (inactivates). When cofilin is turned off, actin polymerization increases, which helps the cell membrane edge generate lamellar and filamentous pseudopodia. This change in the cytoskeleton makes glioblastoma cells far more mobile, invasive, and likely to spread to other parts of the body. Following SRSF1 knockdown, MYO1B redistributes from membrane-associated clusters to a dispersed cytoplasmic pattern. MYO1B AS correlates with SRSF1 levels and predicts poor prognosis (164). In conclusion, the overexpression of SRSF1 causes oncogenic splicing changes in MYO1B, which causes MYO1B to build up at the cell membrane. It acts as a scaffold protein and brings in both PDK1 and PAK1 at the same time, which helps tumors survive, invade, and spread to other parts of the body.

Glioblastoma multiforme (GBM) is the most aggressive type of brain tumor, and it has a terrible prognosis (180–182). Increased angiogenesis is a major sign of GBM. It is marked by the growth of many small blood vessels and the presence of aberrant blood vessels. There is a lot of vascular endothelial growth factor (VEGF) in the GBM microenvironment. In tumor or endothelial cells, the VEGF signaling pathway is quite active (183–185). The VEGF signaling cascade enhances the production and function of the downstream principal transcription factor c-Myc. Activated c-Myc directly binds to the promoter region of the MYO1b gene, acting as a transcriptional activator. This enzyme causes a lot of MYO1b mRNA to be made and the protein to be made too much. Piezo1 is a cation channel that is sensitive to mechanical stress and is in charge of sensing the mechanical stress on the cell membrane. When MYO1b is overexpressed, it changes the local tension and biomechanical properties of the cell membrane by changing the way the cortical actin network is built. MYO1b serves as a “mechanical conduction bridge” beneath the cell membrane. Overexpressing it makes the pulling or stabilizing impact on the cell membrane stronger, which makes it easier for mechanical force to activate the Piezo1 channel (or keeps it open or increases its abundance in the membrane). When Piezo1 is turned on, a lot of calcium ions from outside the cell come in. The rise in calcium signaling inside cells is a major reason why endothelial cells grow, move, and form tubes. This step speeds up the creation of microvessels in glioblastoma, which gives the tumor enough oxygen and nutrients to thrive. The abnormally high levels of MYO1b, a myosin protein, in GBM (glioblastoma) are linked to its malignancy and angiogenesis density, which means that patients have a bad prognosis (186). Because anti-VEGF medicines like bevacizumab do not work very well for treating GBM, targeting the downstream MYO1b or disrupting the MYO1b–Piezo1 interaction could be a new way to stop glioblastoma angiogenesis and get around anti-angiogenic resistance.

#### 5.1.5 Cervical cancer

Cervical cancer (CC) ranks as the third most common cancer globally and the fourth leading cause of cancer death among women worldwide (187). Researchers compared MYO1B expression at both mRNA and protein levels across four CC cell lines (C33A, CaSki, SiHa, and HeLa) and two squamous cell carcinoma (SCC) cell lines (Cal27 and SCC9). Protein expression levels of MYO1B were significantly higher in HPV-positive cells (CaSki, SiHa, and HeLa) compared to HPV-negative cells, indicating MYO1B upregulation in CC tissues and cell lines (188). Based on TCGA and GTEx databases, the prognostic impact of MYO1B mRNA expression was analyzed and evaluated. Kaplan–Meier overall survival curves indicated that high MYO1B mRNA expression correlates with poor survival in patients with CC. Furthermore, inhibiting MYO1B suppressed the growth of CC tumors *in vivo (*188, 189).

Matrix metalloproteinases (MMPs), particularly MMP1/MMP9, are upregulated in cancer tissues and play a key role in human cancer progression (190–193). Following MYO1B knockdown, MMP1/MMP9 mRNA and protein levels were significantly reduced. A positive correlation between MMP1 and MMP9 mRNA expression levels and MYO1B was confirmed in GEPIA’s CC tissues (188). The oncogene HPV16 (the most prevalent HR-HPV) expressed in CaSki and SiHa cells may account for increased MYO1B expression. HPV16 E6/E7 likely regulates MYO1B expression by elevating c-MYC.

Previous studies have demonstrated that inhibiting glycolysis suppresses tumor growth and enhances radiosensitivity in CC cells, suggesting that the extracellular signal-regulated kinase (ERK) pathway may play a crucial role in CC tumorigenesis (194, 195). The ERK pathway can activate the central regulator HIF-1α, which encodes genes for glycolytic enzymes and enzymes converting glucose to lactate, leading to increased aerobic glycolysis (196, 197). Studies indicate that overexpressed MYO1B promotes tumor progression and enhances CC cell migration, invasion, and glycolysis by activating the ERK pathway. MYO1B activation of the ERK1/2 pathway elevates expression levels of HIF-1α and its targets [GLUT1 and lactate dehydrogenase A (LDHA)]. Inhibiting the ERK/HIF-1α pathway in MYO1B-overexpressing cells suppresses their invasive and migratory capabilities (198). Although studies preliminarily reveal MYO1B’s potential role in CC glycolysis, the intrinsic mechanisms underlying MYO1B activation of the ERK pathway warrant further investigation. Furthermore, the specific mechanisms by which MYO1B participates in CC glycolysis require clarification, and its relationship with angiogenesis, drug resistance, and radiotherapy resistance warrants further investigation. In summary, this study identifies a novel potential therapeutic target for CC treatment and reveals a novel function of MYO1B in regulating glucose metabolism.

#### 5.1.6 Colorectal cancer

CRC is the third most common type of cancer worldwide and the second leading cause of cancer-related deaths (199). Many studies have shown that tumor metastasis is the main cause of poor prognosis in patients with CRC (199–204). Previous studies have found that MYO1B is commonly overexpressed in CC, prostate cancer, and head and neck cancer, significantly increasing the risk of tumor metastasis by promoting cancer cell migration, invasion, and motility (146, 170, 188). The expression of MYO1B was increased in most CRC tissues and was positively associated with a greater risk of tumor metastasis and poor prognosis for patients. MYO1B was significantly associated with the migration and invasion properties of CRC cells *in vitro* and *in vivo*. MYO1B also promoted the assembly of focal adhesions by targeting RhoA (56). Further studies indicate that MYO1B inhibits fusion between autologous cells and solutes while enhancing VEGF secretion in CRC cells to promote angiogenesis. Mechanistically, MYO1B blocks the autophagic degradation of HIF-1α, leading to its accumulation and subsequent increased VEGF secretion, which then drives tumor angiogenesis in CRC (205).

Surgery following preoperative chemoradiotherapy (pCRT) is the standard treatment for locally advanced rectal cancer (LARC) (206, 207). Studies investigating whether specific gene and miRNA expression correlates with tumor response to pCRT have identified eight transcripts—TMEM188, ITGA2, NRG, TRAM1, BCL2L13, MYO1B, KLF7, and GTSE1—as strong predictors of tumor response (208).

#### 5.1.7 Neuroblastoma

Neuroblastoma remains the most frequent extracranial solid malignancy in children, responsible for roughly 13% of pediatric cancer mortality (209–211). While MYCN amplification (MNA) is a well-known hallmark of high-risk, aggressive disease, recent data suggest that MYO1B plays a distinct role in this context. Regulated by GREB1 through a MYCN-independent mechanism, high MYO1B expression is frequent in MNA tumors and correlates with poor clinical outcomes (209–217). MYO1B appears to act as more than a driver of invasion; it functions as a key regulator of the secretome. By controlling the extracellular release of the cytokine MIF, MYO1B likely cooperates with MYCN—which drives MIF expression—to fuel tumor progression. This suggests that MNA+ neuroblastoma relies on a complex interplay between MYCN-dependent and -independent pathways.

#### 5.1.8 Breast cancer

Breast cancer (BC) is one of the most frequent type of cancer that affects and kills women (218). It is also the second leading cause of cancer-related death in women. Although surgery, chemotherapy, and radiotherapy can be effective, recurrent treatment resistance and widespread metastasis increase mortality and worsen prognosis (219). In BC, aberrant protein isoform events (AS events) drive disease progression and metastasis, which are critical determinants of patient survival (220). Abnormal AS may cause the production of protein isoforms that promote cell growth, prevent cell death, and increase the ability to spread to other parts of the body. Research indicates that cyperotundone (CYT) disrupts the conventional regulation of MYO1B precursor mRNA splicing by SRSF1 through the downregulation of SRSF1 expression. This lowers the quantity of MYO1B protein, which stops BC cells from proliferating (particularly those that do not respond to drugs) and makes them more responsive to chemotherapy drugs like doxorubicin (221).

#### 5.1.9 Lung adenocarcinoma

Lung adenocarcinoma (LUAD) is the predominant histological subtype of non-small cell lung cancer (NSCLC), accounting for approximately 40% of lung malignancies (222). Circular RNAs (circRNAs) are generated through reverse splicing of precursor mRNAs and regulate biological processes including cancer metabolism (223). Because of their circular structure, circRNAs exhibit greater stability than linear RNAs and hold promise as disease biomarkers. CircRNAs exert their effects through mechanisms such as miRNA sponging, interactions with diverse proteins, cap-independent translation, and splicing regulation (224).

Studies show that when LUAD cells are under energy stress (such as when they do not have enough glucose), they overexpress CircZFR. CircZFR goes into the cell nucleus instead of acting like a sponge for miRNAs, which are small non-coding RNA molecules that regulate gene expression. In the nucleus, CircZFR physically attaches to the splicing factor SRSF1, bringing SRSF1 to certain parts of the MYO1b precursor mRNA (pre-mRNA). This interaction encourages the retention of MYO1b exon 23, resulting in the full-length, functional MYO1b-201 isoform rather than shortened or non-functional versions. The functional MYO1b protein that comes out of this is very important in the cytoplasm, especially near mitochondria. The MYO1b protein interacts directly with the MCU complex, which is on the inner mitochondrial membrane. MARCH5 is an E3 ubiquitin ligase that normally adds ubiquitin to MCU, which causes it to break down. By binding to MCU, MYO1b protects it by keeping MARCH5 from interacting with it in space. This stops MCU from being ubiquitinated and broken down, which makes MCU proteins much more stable and plentiful. Higher levels of MCU make mitochondria take in more calcium (mitochondrial calcium uptake), which raises the amount of Ca^2+^in the mitochondrial matrix. This procedure turns on important dehydrogenases in the tricarboxylic acid (TCA) cycle, namely, PDH, IDH3, and OGDH, which raises the levels of oxidative phosphorylation (OXPHOS) (225). The resulting increase in ATP production supports tumor cell survival under glucose-limited conditions. High levels of MYO1b expression, especially in the subtype containing exon 23, link to a bad prognosis in lung cancer, suggesting it could be a good target for treatment.

Extracellular vesicles (EVs) produced from tumors may function as potential biomarkers for malignancy (226–228). Previous studies have shown that ubiquitin-specific protease 22 (USP22) helps lung cancer spread in both living and nonliving environments (229). Studies demonstrate that USP22 is a deubiquitinating enzyme (DUB) that breaks down K48-linked polyubiquitin chains that are attached to MYO1b. By taking away K48 ubiquitin chains, USP22 blocks MYO1b from breaking down through the ubiquitin–proteasome system (UPS). This makes the MYO1b protein last much longer in cells, which makes the amount of MYO1b protein (not mRNA) go up in cells that make too much USP22. This helps bring multivesicular bodies (MVBs) to the plasma membrane, which makes it more likely that EVs will be released (230). These EVs have signals that make it easier for lung cancer cells to migrate and invade.

### 5.2 Other diseases

#### 5.2.1 Entamoeba histolytic

Thus far, only calcium-binding proteins (CaBPs)—EhCaBP3 and EhCaBP5—have been verified to interact with Eh myosin IB, and they do not engage with the SH3 domain of Eh myosin IB (231). The SH3 domain of Eh myosin IB interacts with the C-terminal domain of EhFP10 (a Rho guanine nucleotide exchange factor) and inhibits its actin-binding activity (232). Calcium signaling is necessary for the start of phagocytosis. This research developed a molecular model: fluctuations in intracellular calcium ion concentration activate EhCaBP5, which subsequently recruits MYO1B to the membrane to commence phagocytosis. If the association between EhCaBP5 and MYO1B is inhibited, or if the function of EhCaBP5 is compromised, MYO1B cannot be efficiently relocated to phagocytic sites, resulting in a substantial reduction in phagocytic efficiency. This shows that moving MYO1B around is a well-controlled “recruitment” process. MYO1B can only change from an inactive or nonspecific state to an active state that is involved in specific phagocytic activities by moving around in this way. The repositioning of MYO1B is not merely a “drift” to the membrane edge, but rather its “anchoring” within specific lipid microenvironments (regions rich in PI3P) through binding to EhFP10. This implies that MYO1B’s repositioning exhibits lipid specificity, aiding in the recognition of phagocytic sites.

At the cell front, PI3K is stimulated, leading to localized accumulation of PI(3,4,5)P3. Phospholipids can induce actin polymerization at the leading edge through small GTPases Cdc42 and Rac, PAKs, and the guanosine triphosphate (GTP) exchange factor PIX, forming new sites. Concurrently, PTEN (a tumor suppressor phospholipase inhibiting the phosphoinositide-3 kinase/Akt pathway) transiently dissociates from the membrane and accumulates at the cell rear. During chemotaxis, the rearward relocalization of PAKa and PTEN appears to facilitate actin complex activation (233–236). In resting parasites, myosin IB localizes to the cytoplasm and accumulates at the cell cortex. During dissemination and phagocytosis in human host intestinal tissue, *Escherichia coli* becomes polarized, with myosin IB relocalizing to pseudopodia where the actin filament network exists in a gel-like state (237).

During erythrocyte phagocytosis, the inner amoeba’s myosin IB is localized within the phagocytic cup from initiation to closure, while its light chain CaBP5 departs after cup closure. Overexpression of myosin IB impairs the inner amoeba’s phagocytic capacity, whereas downregulation of its light chain CaBP5 similarly reduces phagocytic function (231, 237).

#### 5.2.2 Diabetes

Insulin trafficking is facilitated by the actin cytoskeleton, with several members of the extensive myosin superfamily of actin-associated molecular motors implicated in this process (120, 121, 123). MYO1B was expressed in pancreatic β cells and in cultured insulinoma 832/13 cells. MYO1B is localized to the TGN in HeLa cells, and its removal hinders the export of the lysosomal marker MPR from the Golgi apparatus. MYO1B facilitates the development of MPR-positive membrane tubules at the TGN (46). The absence of MYO1B influences an initial phase in the development of nascent (pro)insulin granules. The reduction of MYO1B impairs the first transit of newly produced insulin granules into the transgalactosylating network. The reduced quantity of newly produced granules in MYO1B-depleted 832/3 cells may suggest that the budding of immature insulin granules is compromised without MYO1B. The elimination of MYO1B did not affect actin patches in the perinuclear area of 832/13 cells.

MYO1B was found in proximity to actin patches, the TGN marker TGN38, and insulin granules in the perinuclear region of cultured rat insulinoma 832/13 cells. Depletion of MYO1B via small interfering RNA in 832/13 cells diminished intracellular proinsulin and insulin levels, impaired glucose-stimulated insulin secretion (GSIS), and resulted in the buildup of (pro)insulin secretory granules (SGs) at the TGN. Employing an *in situ* fluorescent pulse-chase methodology to monitor nascent proinsulin, the reduction of MYO1B in insulinoma cells decreased the quantity of (pro)insulin-containing SGs emerging from the TGN. The research demonstrates for the first time that MYO1B regulates GSIS in pancreatic β cells, at least partially by facilitating an initial phase of insulin granule transport from the TGN (238) (as shown in **Table 2**).

**Table 2:** Comprehensive summary of expression patterns, biological functions, molecular mechanisms, and clinical significance of MYO1B across various diseases.

Disease name	Mechanism	Role of MYO1B	References
Head and neck squamous cell carcinoma (HNSCC)	1. PI3K/AKT pathway: MYO1B activates this pathway, promoting ATM phosphorylation and enhancing DNA damage response (DDR). 2. miRNA regulation: Regulated by miR-145 (tumor suppressor) and miR-363; induced by HPV-associated miR-16. 3. Stemness maintenance: Upregulates SOX2/OCT 4. SMAD3/Wnt pathway: Potentially enriched in relationship with SMAD3 and the Wnt pathway.	1. Promotes metastasis: Localizes to protrusions (e.g., filopodia), promoting cell migration and invasion; positively correlated with lymph node metastasis. 2. Radio-resistance: Causes resistance to radiation therapy by accelerating DNA repair and maintaining tumor stem cell characteristics (G0 phase). 3. Biomarker: A potential diagnostic biomarker for predicting lymph node metastasis and prognosis.	(88, 135–156)
Esophageal squamous cell carcinoma (ESCC)	1. miRNA regulation: Directly negatively regulated by miR-145-3p. 2. SNAI2/Cyclin D1 pathway: MYO1B acts as an upstream effector activating this pathway. 3. Splicing regulation: SRSF1 regulates MYO1B distribution from the membrane to the cytoplasm. 4. Ubiquitination: ASB13 may participate in regulating the MYO1B/SNAI2 pathway.	1. Promotes invasion: Overexpression enhances cancer cell invasiveness. 2. Drives tumorigenesis: Drives tumorigenesis and cisplatin cytotoxicity via the SNAI2/cyclin D1 pathway. 3. Prognostic relevance: Positively correlated with high SNAI2 expression, indicating poor prognosis.	(133, 157–169)
Prostate cancer	EMT regulation: Knockdown changes the expression of key regulators of endothelial–mesenchymal transition (EndoMT).	1. Promotes metastasis: Elevated levels in cells with high metastatic potential and metastatic tumors. 2. Biomarker: An indicator of lymph node metastasis in primary tumors.	(170, 171)
Glioma (GBM)	1. Alternative splicing: SRSF1 promotes MYO1B exon 23 retention, forming a full-length isoform acting as a scaffold. 2. PDK1/AKT and PAK1/LIMK pathways: Recruits PDK1 to activate AKT (anti-apoptosis); recruits PAK1 to activate LIMK/Cofilin (cytoskeleton remodeling/pseudopodia). 3. VEGF/c-Myc/Piezo1 axis: VEGF activates c-Myc to upregulate MYO1B; MYO1B opens Piezo1 calcium channels via mechanical force, promoting angiogenesis.	1. Promotes proliferation and survival: Inhibits apoptosis via the AKT pathway. 2. Enhances invasiveness: Promotes pseudopodia formation via actin remodeling. 3. Promotes angiogenesis: Enhances Piezo1-mediated calcium influx, promoting microvessel formation and resistance to anti-angiogenic drugs (e.g., bevacizumab).	(164, 172–186)
Cervical cancer	1. HPV/c-MYC axis: HPV16 E6/E7 upregulates MYO1B by elevating c-MYC. 2. ERK/HIF-1α pathway: Activates ERK1/2, upregulating HIF-1α and its targets (GLUT1, LDHA) to promote glycolysis.	1. Promotes glycolysis: Enhances glucose metabolism to support tumor growth. 2. Promotes migration/invasion: Regulates MMP1/MMP9 expression. 3. Poor prognosis: High expression correlates with low patient survival rates.	(187–198)
Colorectal cancer (CRC)	1. RhoA signaling: targets RhoA to promote focal adhesion assembly. 2. Autophagy regulation: Blocks autophagic degradation of HIF-1α, leading to accumulation and increased VEGF secretion.	1. Promotes metastasis: Enhances cell migration, invasion, and motility. 2. Promotes angiogenesis: Via increased VEGF secretion. 3. Treatment predictor: A strong predictor of tumor response to preoperative chemoradiotherapy (pCRT).	(56, 146, 170, 188, 199–208)
Neuroblastoma	GREB1 regulation: Regulated by GREB1 (MYCN-independent mechanism), but cooperates effectively with MYCN.	1. Secretome regulation: Controls extracellular release of the cytokine MIF, fueling tumor progression. 2. Poor prognosis: High expression correlates with poor clinical outcomes.	(209–217)
Breast cancer	SRSF1 splicing regulation: SRSF1 regulates MYO1B pre-mRNA splicing; this axis is disrupted by the drug cyperotundone (CYT).	1. Promotes proliferation: Aberrant isoforms promote cell growth. 2. Drug resistance: Knockdown enhances sensitivity to chemotherapy drugs like doxorubicin.	(218–221)
Lung adenocarcinoma	1. CircZFR/SRSF1 axis: Energy stress triggers CircZFR to recruit SRSF1, promoting MYO1B exon 23 retention. 2. Mitochondrial metabolism: Full-length MYO1B protects the MCU complex from MARCH5-mediated ubiquitination, increasing mitochondrial calcium uptake and activating TCA cycle/OXPHOS. 3. USP22 deubiquitination: USP22 removes ubiquitin chains from MYO1B, stabilizing the protein and promoting extracellular vesicle (EV) release.	1. Metabolic adaptation: Generates ATP during glucose deprivation to support survival. 2. Promotes metastasis: Facilitated by the release of EVs carrying invasive signals. 3. Poor prognosis: High expression correlates with poor outcomes.	(222–230)
Amoebiasis (*Entamoeba histolytica*)	Calcium signaling/lipid binding: Intracellular calcium activates EhCaBP5, recruiting MYO1B to PI3P-rich membrane regions (phagocytic cup); binds to EhFP10.	1. Phagocytosis: Acts as a molecular motor providing tension to close the phagocytic cup. 2. Motility: Participates in pseudopodia formation and parasite migration through tissues.	(231–238)
Diabetes	Golgi trafficking: Localizes to the trans-Golgi network (TGN) and interacts with actin.	1. Insulin secretion: Regulates glucose-stimulated insulin secretion (GSIS). 2. Granule formation: Facilitates the initial budding and transport of insulin secretory granules from the TGN.	(46, 120–123)

## 6 Conclusion and prospects

MYO1B is a single molecule of class I myosin that works as a mechanochemical transducer at the membrane–cytoskeleton interfaces. It generates force by breaking down ATP, which controls the tension in the membrane and the turnover of actin. This is different from transport motors. MYO1B is essential for structuring the cortical cytoskeleton to facilitate cell motility and endocytosis. It also modifies organelles by changing how actin moves around in the TGN and endolysosomal system. This controls vesicle trafficking and important cellular morphogenesis.

MYO1B functions as a *bona fide* driver of pathogenesis by coupling cytoskeletal architecture with downstream signal transduction, rather than acting solely as a passive transport motor. In colorectal carcinoma, for instance, MYO1B-mediated activation of the ROCK2/LIMK/Cofilin axis directly governs the actin dynamics required for invasion. Distinct from this cytoskeletal role, MYO1B modulates the tumor microenvironment in neuroblastoma via a GREB1–MYO1B–MIF secretory axis. This regulatory function encompasses metabolic homeostasis, wherein the spatial restriction of lysosomes by MYO1B governs mTORC1-S6K1 signaling efficacy and the ensuing vascular aging. Thus, MYO1B dysregulation constitutes a central, context-dependent mechanism promoting malignant and metabolic phenotypes.

Dissecting the causal role of MYO1B requires moving beyond correlative expression profiles to isolate specific molecular activities—specifically motor torque generation and lipid binding. Current loss-of-function models are frequently confounded by functional compensation among class I paralogs, obscuring the mechanotransduction steps that link membrane tension to MAPK/mechanistic target of rapamycin kinase (mTOR) activation. Resolution of these pathway dynamics necessitates *in vivo* separation-of-function mutants and the mapping of compartment-specific interactomes at the TGN and endosomes. Such mechanistic precision is critical to determine if MYO1B inhibition can selectively suppress malignancy while preserving basal homeostatic trafficking.

Associative evidence linking MYO1B to oncogenic pathways largely obscures the causal hierarchy: does pathology arise from stochastic motor activity or specific lipid-sorting events? The lack of a cohesive model is exacerbated by compensatory mechanisms within the myosin family that mask the effects of broad ablation strategies. Furthermore, the mechanochemical interface—the translation of physical actin-membrane coordination into kinase signaling cascades—lacks structural and spatiotemporal resolution. Future dissection must therefore prioritize the uncoupling of motor torque from membrane anchoring. By utilizing rigorous separation-of-function mutants rather than simple knockdowns, we can define the precise biophysical sequence governing recruitment to the TGN or endosomes. This granularity is essential to determine if MYO1B inhibition can selectively target malignant phenotypes without collapsing the essential housekeeping functions of intracellular transport.

## Glossary

ADP: Adenosine diphosphate
AMPK: AMP-activated protein kinase
AS: Alternative splicing
ASB13: Ankyrin repeat and SOCS box containing 13
ATP: Adenosine triphosphate
CaBPs: Calcium-binding proteins
CDPK: Calcium-dependent protein kinase
CC: Cervical cancer
CDC: Cell division cycle
CRC: Colorectal cancer
DHRS2: Dehydrogenase/reductase family member 2
EGFP: Enhanced green fluorescent protein
Eh: Entamoeba histolytica
EMT: Epithelial–mesenchymal transition
ERK: Extracellular signal-regulated kinase
ESCC: Esophageal squamous cell carcinoma
F-actin: Filamentous actin
FAK: Focal adhesion kinase
GREB1: Growth regulating estrogen receptor binding 1
GSIS: Glucose-stimulated insulin secretion
GTP: Guanosine triphosphate
HCM: Hypertrophic cardiomyopathy
HNSCC: Head and neck squamous cell carcinoma
I/R: Ischemia/reperfusion
IHC: Immunohistochemistry
Kif5C: Kinesin family member 5C
LAH: Lever arm helix
LARC: Locally advanced rectal cancer
LCBD: Light-chain binding domain
LDHA: Lactate dehydrogenase A
LIMK: LIM domain kinase
LNM: Lymph node metastasis
LRRK2: Leucine-rich repeat serine/threonine-protein kinase 2
MDM2: Mouse double minute 2
MIF: Migration inhibitory factor
MMPs: Matrix metalloproteinases
MPR: Mannose-6-phosphate receptor
mTOR: Mechanistic target of rapamycin kinase
MYO: Myosin
NB: Neuroblastoma
NCD: Non-claret disjunctional
NTR: N-terminal region
OTSCC: Oral tongue squamous cell carcinoma
PH: Pleckstrin homology domain
Pi: Inorganic phosphate
PI3K: Phosphatidylinositol-3 kinase
PKB: Protein kinase B
PPIs: Protein–protein interactions
PTEN: Phosphatase and tensin homolog
RhoA: Ras homolog family member A
ROCK: Rho-associated coiled-coil containing protein kinase
SCC: Squamous cell carcinoma
SGs: Secretory granules
SH: Src homology domain
SNX: Sorting nexin
SRSF1: Serine/arginine-rich splicing factor 1
TGN: Trans-Golgi network
TH: Tyrosine hydroxylase
